# Prognostic value of genomic damage in non-small-cell lung cancer.

**DOI:** 10.1038/bjc.1998.327

**Published:** 1998-06

**Authors:** C. de Juan, P. Iniesta, F. J. Vega, M. A. Peinado, C. Fernandez, T. CaldÃ©s, M. J. Massa, J. A. LÃ³pez, A. SÃ¡nchez, A. J. Torres, J. L. Balibrea, M. Benito

**Affiliations:** Departamento de BioquÃ­mica y BiologÃ­a Molecular, Facultad de Farmacia, Universidad Complutense, Madrid, Spain.

## Abstract

**Images:**


*British Journal of Cancer* (1998) **77**(11), 1971–1977
© 1998 Cancer Research Campaign

### Prognostic value of genomic damage in non-small-cell lung cancer

**C de Juan[1], P Iniesta[1], FJ Vega[1], MA Peinado[2], C Fernandez[3], T Caldés[4], M José Massa[1], JA López[5], A Sánchez[6], AJ Torres[6], JL Balibrea[6] and M Benito[1]**

[1]Departamento de Bioquímica y Biología Molecular, Facultad de Farmacia, Universidad Complutense, 28040 Madrid, Spain; [2]Institut de Recerca Oncologica, L'Hospitalet, 08907 Barcelona, Spain; Servicios de [3]Medicina Preventiva, [4]Inmunología, [5]Anatomia Patológica and [6]Cirugía, Hospital Universitario San Carlos, 28040 Madrid, Spain

**Summary** Genomic alterations have been analysed in 65 non-small-cell lung cancer (NSCLC) tissue samples by using the arbitrarily primed polymerase chain reaction (AP-PCR), which is a PCR-based genomic fingerprinting. We have shown that AP-PCR may be applied as a useful and feasible practical method for detection of the genomic alterations that accompany malignancy in NSCLC. Genomic changes detected by us consisted of: allelic losses or gains in anonymous DNA sequences, homozygously deleted DNA sequences and polymorphic DNA sequences. According to these genomic changes, lung tumours evaluated in the present study have been scored into three groups: low, moderate and high genomic damage tumours. The aim of this study was to investigate the effect of genomic damage on patient survival. Survival analysis was carried out in 51 NSCLC patients. Our results revealed that high genomic damage patients showed a poorer prognosis than those with low or moderate genomic damage ($P = 0.038$). Multivariate Cox regression analysis showed that patients with higher genomic alterations displayed an adjusted-by-stage risk ratio 4.26 times higher than the remaining patients (95% CI = 1.03–17.54). We can conclude that genomic damage has an independent prognostic value of poor clinical evolution in NSCLC.

**Keywords:** genomic damage; non-small-cell lung cancer; arbitrarily primed PCR; prognostic value

Cancer is the result of the accumulation of multiple genetic changes in the tumour cell genome. Each alteration, whether an initiating or a progression-associated event, may be mediated through a gross chromosomal change and therefore has the potential to be cytogenetically visible.

Several molecular genetic methods have been applied to detect those genomic alterations in tumour DNAs. Arbitrarily primed polymerase chain reaction (AP-PCR), a genomic fingerprinting method (Welsh and McClelland, 1990; 1991), has become established, in the last few years, as an efficient screening method to detect novel genetic alterations in cancer cells (Peinado et al, 1992; Khono et al, 1994; Achille et al, 1996; Okazaki et al, 1996; Vogt et al, 1996). The method relies on the selective amplification of genomic sequences that, by chance, are flanked by adequate matches to a primer whose nucleotide sequence is arbitrarily chosen. Tumour-specific somatic genetic alterations can be readily detected by comparison of the AP-PCR fingerprints from tumoral and normal tissue of the same patient. Peinado et al (1992) applied this fingerprinting method to analyse genetic alterations in colorectal carcinomas and demonstrated that the decreased and increased intensities of the PCR bands in tumour DNA represent allelic losses and gains, respectively, of the corresponding genomic fragments in cancer cells. Other investigators have identified a novel chromosomal locus homozygously deleted in a human small-cell lung cancer (SCLC) cell line using AP-PCR genome fingerprinting (Kohno et al, 1994). In other studies, AP-PCR has been applied for detection of amplified genomic sequences in human SCLC cell lines (Okazaki et al, 1996).

*Received 12 February 1997*
*Revised 22 October 1997*
*Accepted 30 October 1997*

*Correspondence to:* M Benito

In this investigation, we have undertaken a detailed AP-PCR study to detect genomic alterations in non-small-cell lung carcinomas (NSCLCs). NSCLC originates mainly from bronchial epithelial cells and represents the majority of human lung cancers. These carcinomas are surgically managed if both the stage at which diagnosis is made and the clinical status are appropriate. Genomic DNA from 65 NSCLC patients undergoing radical surgery was analysed by comparing DNA fingerprinting from tumour tissues and their corresponding normal tissues. Changes observed in tumour cells, including increases and decreases in the relative intensity of the tumour band compared with the normal control band, were considered.

The aims of this study were, first, to establish if AP-PCR technique could be important for analysis of the cancer genome and, second, to investigate if a higher accumulation of genomic alterations detected by this method, could be associated with a poor clinical evolution in NSCLC patients. In addition, we tried to establish a relationship between AP-PCR genomic alterations and other molecular changes considered to be most prevalent in lung tumours, such as K-*ras* and p53 mutations (Slebos et al, 1990; Rodenhuis and Slebos, 1992; Horio et al, 1993; Mitsudomi et al, 1993; Vega et al, 1996; 1997), as well as c-*myc* overexpression, considered as a relatively late event in the lung cancer development (Bergh, 1990). Our results suggest that AP-PCR may be applied as a useful and feasible practical screening method for detection of novel somatic genomic alterations in NSCLC and could be used with a diagnostic and/or prognostic purpose.

### MATERIALS AND METHODS

### Patients and tumour samples

Non-small-cell lung carcinomas and corresponding normal tissues were obtained from 65 patients (64 men and 1 woman) with a median age of 62.2 ± 9.25 years, who underwent surgery between 1992 and 1994 at the San Carlos Hospital (Madrid, Spain). No specific decision was taken to restrict this study to male patients; patients were included in a consecutive way. During surgery, two tissue samples were obtained: from the tumoral specimen and from normal lung parenchyma (at least 4 inches away from the distal margin of the neoplasm). All tissue samples were snap-frozen in liquid nitrogen immediately after surgery and stored at –80°C. Cryostat-sectioned, haematoxylin and eosin-stained samples from each tumour block were examined microscopically by two independent pathologists to confirm the presence of > 80% tumour cells. Paired normal tissues from the same individual were used as control.

Histological classification of the tumours was based on the WHO classification (WHO, 1981). The distribution of the histological types was 40 (61.5%) squamous cell carcinomas (SCC), 16 (24.6%) adenocarcinomas (AC) and nine (13.8%) large cell undifferentiated carcinomas (LCUC). According to differentiation grade, eight (12.3%) tumours were scored as well differentiated, 31 (47.7%) moderately and 26 (40%) poorly differentiated. Tumours were pathologically staged using the tumour node metastasis (TNM) system (Mountain, 1986) and consisted of 30 (46.1%) stage I tumours, five (7.7%) stage II, 23 (35.4%) stage $III_A$, three (4.6%) stage $III_B$ and four (6.2%) stage IV.

Patients who had stage I, II and $III_A$ tumours were subjected to curative surgery, whereas only a biopsy was taken from patients who suffered greater disease extension (tumours in stages $III_B$ and IV). Patients with resected tumour were seen at 3-month intervals during the first 3 years of follow-up in order to perform clinical examination, chest radiography and serum tumour marker analysis. Bronchoscopy, and thorax and upper abdominal (CT) computerized tomography were performed twice a year. During the next 2 years, visits and exploration were reduced to half.

### DNA isolation

Genomic DNA from all samples was prepared by proteinase K and the phenol–chloroform extraction method as described previously (Blin and Stafford, 1976).

### AP-PCR

AP-PCR is a PCR-based method for DNA fingerprinting (Welsh and McClelland, 1990; 1991). Amplifications, using a single arbitrary primer, were performed under low-stringency conditions in the initial cycles in order to hybridize the arbitrary primers to many sequences in the total genomic DNA. Then, following cycles under high-stringency conditions allow only the best matches of the initial events to be amplified further.

Genomic DNA (25–50 ng) was incubated with 0.75 units of *Taq* DNA polymerase (Perkin Elmer, Roche, NJ, USA) and 1 unit of *Taq* DNA polymerase Stoffel fragment (Perkin–Elmer, Roche, NJ, USA), 200 μM of each dNTP (Pharmacia, Biotech, Uppsala, Sweden), 1–2 μCi of [α-$^{32}$P] dCTP (3000 Ci mmol$^{-1}$, Amersham, UK), 10 mM Tris-HCl (pH 8.3), 50 μM potassium chloride, 2.5 mM magnesium chloride and 0.5 μM of an arbitrary primer, in a volume of 25 μl. Primer sequences were as follows: 5–6a: 5′-AGTTG-CAAACCAGACCTCAG-3′; 6s: 5′-CACTGATTGCTCTTAGGT-CTG-3′; 6i: 5′-TCTTAGGTCTGGCCCCTCCT-3′.

**Figure 1** AP-PCR analysis of non-small-cell lung tumours. Autoradiograms of $^{32}$P-labelled DNA fragments amplified by AP-PCR are shown. Total genomic DNA (50 ng) from normal (N) and tumoral (T) tissue pairs of the patients indicated at the top was amplified with primers 6i (A) and 5–6a (B). Increases and decreases in the intensity of tumour bands are indicated with arrowheads and denominations as used in the text

Primer selection was completely arbitrary, this means that there was no intentional bias in favour or against any type of sequence. We evaluated single primers that had been previously used in our laboratory for other studies. Those primers generating an adequate amplification pattern from genomic DNA were chosen for the AP-PCR experiments.

Amplification reactions were carried out in a 2400 GeneAmp PCR system (Perkin–Elmer) for five cycles under low-stringency conditions (95°C for 45 s, 40°C for 30 s and 72°C for 1.15 min) followed by 35 cycles under higher stringency conditions (95°C for 45 s, 60°C for 30 s and 72°C for 1 min). Denatured PCR products were analysed in 6% denaturing polyacrylamide/7 M urea gels

 © Cancer Research Campaign 1998

**Figure 2** AP-PCR DNA fingerprinting of normal (N) and tumoral (T) tissue pairs of the patients indicated at the top, amplified with primer 6s. Polymorphic DNA sequences are indicated on the left with arrowheads and with denominations as used in the text. Sizes of bands, in nucleotides (nt), are indicated on the right. Note that patient 75 is shown in duplicate. Reproducible fingerprints were obtained in both cases at 7 W for 18 h at room temperature. The gels were dried and exposed to radiograph film.

### Quantification of DNA fingerprinting tumour bands

By using AP-PCR, we generated complex DNA fingerprints consisting of 40–50 bands for each patient and for a given primer used. Because of the great number of bands to be analysed by comparing intensity changes between normal and tumour bands, we first selected all the changes detected by two independent observers. After this previous screening, we confirmed and quantified the rates of changes by using a BioImage IQ and TDI IQ+ software (TDI, Spain) for gel electrophoresis. Intra- and interassay reproducibility was assessed. For intra-assay variability, DNA samples were analysed in duplicate, i.e. patient 75 in Figure 2. Results revealed that reproducible DNA fingerprints were obtained in both cases. For interassay reproducibility, ten normal–tumour tissue pairs, randomly selected from the sample collection, were analysed with primers 5–6a and 6s three to five times. Reproducibility analysis indicated that band intensity changes of up to 20% could be produced by interassay variability. In consequence, increased and decreased intensities of tumour bands were only considered when amplification or reduction in signal intensity from tumour tissue, in comparison to the corresponding signal from the normal control tissue, was greater than 20%. Only cases displaying a similar level of amplification (lane overall intensity) of the normal and tumour fingerprints were considered for analysis. Unequal amplification of both lanes could be due to poor DNA quality or reaction failure. Of all the normal–tumour tissue DNA fingerprinting pairs analysed in this study ($65 \times 3 = 195$), only ten (5%) displayed non-reproducible fluctuations in the in vitro amplification. In these cases quantitative changes could be spurious and were not considered for AP-PCR analysis.

### Detection of K-*ras*, c-*myc* and p53 genetic alterations

The detection of K-*ras* point mutations was performed as described by Vega et al (1996). p53 gene mutations were detected as described by Vega et al (1997). Accumulation of the p53 protein in the tumour cell nuclei was detected as described by Vega et al (1997). C-*myc* overexpression was evaluated by Northern blot analysis (unpublished results).

### Statistical analysis

Molecular abnormalities previously investigated in the tumour population analysed in this study, such as K-*ras* mutations, p53 alterations and c-*myc* overexpression, were correlated with data obtained from AP-PCR analysis. Clinicopathological characteristics of tumours analysed were also correlated with results from AP-PCR. Results were evaluated by the chi-square test and a *P*- value < 0.05 was judged to be statistically significant.

Survival analysis was carried out with patients whose clinical evolution was followed up for at least 24 months. We only considered patients with I, II and $III_A$ tumours, and patients who had died in the post-operative period were also excluded. Thus, the number of patients included in the survival study was 51.

Survival curves were calculated using the Kaplan–Meier method and compared using the log-rank test. Results were considered significant for *P*-values < 0.05. The Cox proportional hazards model for univariate analysis was applied to calculate risk ratios and 95% confidence intervals. Multivariate analysis using the proportional hazards model of Cox was used to estimate simultaneously the relative strength of multiple covariates to assess their statistical independence.

### RESULTS

### AP-PCR DNA fingerprinting of non-small-cell lung carcinomas

It was possible to generate reproducible fingerprints of normal–tumour tissue DNA pairs from 65 patients with NSCLC using AP-PCR. Figures 1 and 2 show representative experiments, each obtained using a different arbitrary primer: 6i (Figure 1A), 5–6a (Figure 1B) and 6s (Figure 2), (for sequences see Materials and methods). We analysed 130 samples of genomic DNA (65 normal control and 65 tumour samples). Each normal–tumour tissue pair

© Cancer Research Campaign 1998

Table 1 Relationship between molecular abnormalities and genomic alterations detected using AP-PCR

| Genomic damage | Number of cases | K-*ras* gene mutations number (%) | c-*myc* mRNA overexpression number (%) | p53 protein accumulation number (%) | p53 gene mutations number (%) |
|---|---|---|---|---|---|
| Low | 23 | 5 (21.7) | 5 (21.7) | 12 (52) | 4 (17.4) |
| Moderate | 30 | 8 (26.7) | 11 (36.6) | 17 (56.7) | 7 (23.3) |
| High | 12 | 0 (0) | 5 (41.7) | 8 (66.7) | 4 (33.3) |
| Total | 65 | 13 (20) | 21 (32) | 37 (56.9) | 15 (23) |
| *P*-value | | 0.143 | 0.383 | 0.712 | 0.568 |

was analysed with the arbitrary primers mentioned above. Thus, a total of 390 DNA fingerprints were analysed in this study. For a given primer, band patterns consisted of 40–50 DNA fragments of sizes ranging from 200 to 1000 nt. The average number of DNA bands obtained with the three arbitrary primers used was 138.8 ± 3.3. When we analysed the AP-PCR fingerprints we detected changes in the intensity of several bands. Increased or decreased intensities in tumour bands relative to their corresponding normal bands were observed. For instance, the density of bands D1 and D2 of tumour 50 (Figure 1A) was reduced compared with its normal tissue band. The same tumour also showed some higher molecular weight DNA fragments with a greater density than the normal bands (1, 2 and 3). Other examples of tumour increased bands were bands 14, 15 and 16 of tumour 39 (Figure 1B). This tumour also showed a decreased intensity band in relation to its normal control band (D3). Band P3 of tumour 77 was another apparent case of decreased intensity band (Figure 2). The frequency of the increased intensity tumour bands (gains) was higher than the decreased ones (losses). Sixty-four per cent of all genomic changes analysed by us consisted of gains in the corresponding genomic fragments.

In addition, in some cases we detected changes when comparing DNA fingerprints obtained from different patients. These differences represented polymorphism in the human population because they were present in both normal and tumour tissues from many patients. For instance, band P1 was absent in patients 63, 75 and 16 (Figure 2) and band P2 was also absent in patients 13 and 75 (Figure 2). At the bottom of Figure 2 there was a case of length polymorphism (bands P3 upper and lower). There were some patients with an upper P3 band (87 and 73), patients with a lower P3 band (75, 16 and 77) and other patients showed both of them (11, 13, 63, 69 and 71). These bands appeared to represent two alleles from the same polymorphic locus.

Finally, P3 bands present in normal tissue were apparently absent in tumoral tissue from patients 11 and 71, suggesting that these sequences were homozygously deleted in both of them (Figure 2).

### Quantification of genomic damage

Changes observed in tumoral tissue related to normal tissue were analysed, taking into account increases and decreases in the relative intensity of the tumour bands. For a given patient, the total number of changes detected by AP-PCR was divided by the total number of bands obtained with the three arbitrary primers used in this study (138.8 ± 3.3). For instance, in tumour 50 five changes were detected with primer 6i (three increases and two decreases) (Figure 1A); eight changes with primer 6s (five increases and three decreases); and four changes with primer 5–6a (three increases and one decrease). Thus, the total number of changes detected was 17 and the total number of bands obtained with primers 6i, 6s and 5–6a was 142. The quotient obtained from these two values was taken as the degree of AP-PCR genomic damage for this patient. According to the distribution of the degrees of genomic damage in NSCLC patients, tumours evaluated in the present study were subjectively classified into three groups: low, moderate and high genomic damage tumours. Twenty-three patients (35%) had low damage, 30 (46%) had moderate damage and 12 (18%) had high genomic damage. The first group of tumours showed a number of genomic changes of 5.1 ± 2.5. The degree of genomic damage for these tumours was < 0.07. Moderate genomic damage tumours displayed a number of alterations of 12.6 ± 2.6. In this case the degree of genomic damage ranged between 0.07 and 0.140. Finally, high genomic damage tumours showed an average number of 20.1 ± 3.5 changes and a degree of genomic damage > 0.140.

No significant correlation was found between genomic damage and the clinicopathological characteristics of the tumours analysed, such as tumour stage, histological type and differentiation grade.

**Figure 3** Survival curves using Kaplan–Meier analysis of radically resected NSCLC patients in relation to genomic damage (only patients who had stage I, II and $III_A$ tumours and excluding patients who had died in the post-operative period). The median follow-up period was 110 weeks. High genomic damage patients ( — ) vs low and moderate genomic damage patients (– –), $P = 0.038$ using the log-rank test

 © Cancer Research Campaign 1998

**Table 2** Results of univariate risk estimates for survival according to Cox proportional hazards model for prognostic variables in stages I, II and $III_A$ tumours of NSCLC patients ($n = 51$)

| Factor | Category | Risk ratio | 95% CI | P |
|---|---|---|---|---|
| Genomic damage (AP-PCR) | High vs low + moderate | 2.71 | 0.89–8.23 | 0.10 |
| Stage | II vs I | 6.50 | 1.07–39.26 | |
| | $III_A$ vs I | 6.87 | 1.48–31.93 | 0.01 |
| Histology | Squamous cell vs adenocarcinoma | 1.34 | 0.29–6.24 | 0.70 |
| Differentiation grade | Poor vs well + moderate | 1.37 | 0.47–3.97 | 0.55 |

CI = confidence interval.

**Table 3** Results of multivariate risk estimates for survival according to Cox proportional hazards model for prognostic variables in stages I, II and $III_A$ tumours of NSCLC patients ($n = 51$)

| Factor | Category | Risk ratio | 95% CI | P |
|---|---|---|---|---|
| Genomic damage (AP-PCR) | High vs low + moderate | 4.26 | 1.03–17.54 | 0.04 |
| Stage | II vs I | 3.00 | 0.43–20.74 | 0.27 |
| | $III_A$ vs I | 8.66 | 1.79–41.77 | 0.01 |

CI = confidence interval.

### Genomic alterations detected by AP-PCR in relation to other molecular abnormalities

Previous studies carried out in our laboratory investigated K-*ras*-activating point mutations in a group of NSCLC patients, including all the patients considered in this study. Furthermore, we investigated p53 protein accumulation and p53 gene mutations in all these tumours, as well as c-*myc* overexpression. Taking into account these studies, we have investigated if there was a relationship between the molecular changes mentioned above and the genomic alterations detected by AP-PCR. The results obtained did not show a significant association between these parameters (Table 1).

### Effect of AP-PCR genomic alterations on patient survival

After molecular genomic analysis, a statistical analysis was performed to examine the prognostic significance of genomic alterations detected by AP-PCR in NSCLC patients.

Kaplan–Meier survival curves have shown that patients with high genomic damage have a worse prognosis than those with a low or a moderate level of genomic alterations ($P = 0.038$) (Figure 3). When a univariate Cox regression analysis was performed, the variable stage was the only significant prognostic factor ($P = 0.01$) (Table 2). Patients who had stage II and $III_A$ tumours showed a risk ratio much higher than those who had stage I tumours. The variable genomic damage (AP-PCR) did not reach statistical significance ($P = 0.10$), but the risk ratio of patients with high vs low + moderate genomic damage was 2.71 (95% CI = 0.89–8.23).

To assess the independence of genomic damage and stage as possible prognostic indicators, multivariate Cox regression analysis was performed. As noted in Table 3, patients with high genomic damage showed an adjusted-by-stage risk ratio 4.26 times higher than the remaining patients (95% CI = 1.03–17.54). When stage $III_A$ tumour patients were compared with those in stage I, an adjusted risk ratio of 8.66 (95% CI = 1.79–41.77) was calculated.

### DISCUSSION

In this study, genomic alterations occurring in NSCLC have been analysed using AP-PCR. Genomic damage detected by this technique has been associated with clinical evolution of NSCLC patients. AP-PCR offers immediate application as an alternative approach to examine genomic damage in cancer cells. In the last few years, this DNA fingerprinting method has been successfully applied to the investigation of genomic alterations in colorectal tumours (Peinado et al, 1992), human small-cell lung cancer (SCLC) cell lines (Khono et al, 1994; Okazaki et al, 1996), pancreatic carcinoma (Achille et al, 1996) and skin tumours (Vogt et al, 1996).

In this study, we have shown that AP-PCR is very useful for the detection and the characterization of genomic alterations that accompany malignancy in NSCLC. These abnormalities consisted of: (a) increases or decreases in the intensity of tumour bands relative to normal bands from the same individual; (b) differences because of polymorphism in the human population; and (c) homozygously deleted sequences in some tumour tissues.

(a) Regarding intensity differences in arbitrarily primed PCR tumour bands, previous studies reported by Peinado et al (1992) demonstrated that decreased and increased intensities of the AP-PCR bands in tumour DNA represent allelic losses and gains, respectively, of the corresponding genome fragments in cancer cells. Allelic losses could be due to their linkage to suppressor genes and gains might reflect the presence of extra copies of those sequences, which could be due to gene amplification or to chromosomal imbalance as a result of the tumour cell aneuploidy. The possibility of detecting moderate gains of genetic material, such as those corresponding to triploidy and tetraploidy, represents a significant technical development because such genomic changes cannot be readily detected through conventional allelotyping by restriction fragment length polymorphism or by typing of microsatellites.

© Cancer Research Campaign 1998

We have shown that gains in genomic sequences occur at high frequency (64% of all genomic changes analysed) in non-small-cell lung carcinomas. A frequent presence of amplifications of multiple loci in cancer cell lines and primary tumours has been demonstrated by molecular cytogenetic analysis (Kallioniemi et al, 1992, 1993; Ried et al, 1994). The high proportion of gains in genomic sequences in our lung tumour samples may indicate a main role of moderate increases in gene copy number in NSCLC.

(b) In addition, we have detected another type of change present only in a few of the patients considered in this study, in both normal and tumour tissues. These differences represent polymorphisms in the human population. DNA polymorphism analysis by AP-PCR has been widely used to identify bacterial species and strains as well as plant varieties (Gomez-Lus et al, 1993; Martinez-Murcia et al, 1994; Perolat et al, 1994; Yi et al, 1995).

(c) Finally, we have found homozygously deleted sequences in some tumour tissues. Chromosomal deletions in human tumours have been regarded as evidence that the affected regions contain tumour-suppressor genes (Weinberg, 1991). Several of these genes have been cloned from the regions of homozygous deletions in human cancer (Friend et al, 1986). Previous studies of Khono et al (1994) reported that a homozygous deletion was detected by AP-PCR genomic fingerprinting at chromosome 2q33 in a human SCLC cell line, suggesting the presence of a novel tumour-suppressor gene. These results demonstrated the use of the AP-PCR technique for the detection and characterization of genes that may be involved in lung carcinogenesis.

Considering all the genomic alterations detected in the non-small-cell lung tumours analysed in this study, we have classified them into three different groups according to the higher or lower incidence of these molecular changes. Once these groups of tumours were established, we tried to correlate the incidence of genomic damage detected by AP-PCR with clinicopathological features of tumours, as well as with some prevalent genetic abnormalities previously investigated by us in these lung tumours, such as K-*ras* mutations (Vega et al, 1996), p53 alterations (Vega et al, 1997) and c-*myc* overexpression (unpublished results). When we correlated the incidence of genomic damage with tumour stage, histology and differentiation, our results did not show significant differences. Besides, when we investigated if the incidence of genomic damage had any relationship with the genetic abnormalities mentioned above, our results showed that there was a non-significant association between these parameters. Other significant genetic changes in lung cancer such as 3p deletions, microsatellite instability and changes in telomerase activity should be investigated in relation to genomic damage. At the moment we are performing studies conducted to investigate the relationship between genomic alterations and microsatellite instability. Data obtained from this study would also be useful to recognize if defects in DNA repair pathways constitute a route for human carcinogenesis that predispose to an increase in mutations in the genome.

We have considered an obvious direct application of the AP-PCR technique for cancer prognosis. The association between genomic aberrations detected using this method and patient survival, to our knowledge, has not been described previously in NSCLC. Our results showed a significant poorer prognosis in NSCLC patients with a higher prevalence of genomic alterations. Multivariate Cox regression analysis also showed that tumour genomic alterations and tumour stage could be considered as independent prognostic markers. Taking together all these results, it seems that there is enough evidence to suggest that a high level of genomic alterations may have a predictive prognostic value in NSCLC patients.

In conclusion, our results indicate, first, that AP-PCR is a feasible and useful technique for detection and characterization of novel genomic alterations in NSCLC, and, second, that genomic damage has an independent prognostic value of poor clinical evolution in NSCLC.

### ABBREVIATIONS

NSCLC, non-small cell lung cancer; SCLC, small-cell lung cancer; AP-PCR, arbitrarily primed polymerase chain reaction.

### ACKNOWLEDGEMENTS

The authors gratefully thank Antonio Fernandez (Instituto de Investigaciones Biomédicas, CSIC) for the photographic service. This work was supported by the grants PR 179/91-3472 from Universidad Complutense de Madrid and 94/1557 from FIS (Ministerio de Sanidad y Consumo), Spain.

### REFERENCES

Achille A, Biasi MO, Zamboni G, Bogina G, Magalini AR, Pederzoli P, Perucho M and Scarpa A (1996) Chromosome 7q allelic losses in pancreatic carcinoma. *Cancer Res* **56**: 3808–3813

Bergh JCS (1990). Gene amplification in human lung cancer. *Am Rev Respir Dis* **142**(suppl.): 20–26

Blin N and Stafford DW (1976) A general method for isolation of high molecular weight DNA from eukaryotes. *Nucleic Acids Res* **3**: 2303–2308

Friend SH, Bernards R, Rojelj S, Weinberg RA, Rapaport JM, Albert DM and Dryja TP (1986) A human DNA segment with properties of the gene that predisposes to retinoblastoma and osteosarcoma. *Nature* **323**: 643–646

Gomez-Lus P, Fields BS, Benson RF, Martin WT, O'Connor SP and Black CM (1993) Comparison of arbitrarily primed polymerase chain reaction, ribotyping, and monoclonal antibody analysis for subtyping *Legionella pneumophila* serogroup 1. *J Clin Microbiol* **31**: 1940–1942

Horio Y, Takahashi T, Kuroishi T, Hibi K, Suyama M, Niimi T, Shimokata K, Yamakawa K, Nakamura Y, Ueda Y and Takahashi T (1993) Prognostic significance of p53 mutations and 3p deletions in primary resected non-small cell lung cancer. *Cancer Res* **53**: 1–4

Kallioniemi A, Kallioniemi OP, Sudar D, Rutovitz D, Gray JW, Waldman F and Pinkel D (1992) Comparative genomic hybridization for molecular cytogenetic analysis of solid tumors. *Science* **258**: 818–821

Kallioniemi OP, Kallioniemi A, Sudar D, Rutovitz D, Gray JW, Waldman F and Pinkel D (1993) Comparative genomic hybridization: a rapid new method for detecting and mapping DNA amplifications in tumors. *Sem Cancer Biol* **4**: 41–46

Kohno T, Morishita K, Takano H, Shapiro DN and Yokota J (1994) Homozygous deletion at chromosome 2q33 in human small-cell lung carcinoma identified by arbitrarily primed PCR genomic fingerprinting. *Oncogene* **9**: 103–108

Martinez-Murcia AJ and Rodriguez-Varela F (1994) The use of arbitrarily primed PCR (AP-PCR) to develop taxa especific DNA probes of known sequence. *FEMS Microbiol Lett* **124**: 265–269

Mitsudomi T, Oyama T, Kusano T, Osaki T, Nakanishi R and Shiravakusa T (1993) Mutations of the p53 gene as predictor of poor prognosis in patients with non-small cell lung cancer. *J Natl Cancer Inst* **85**: 2018–2023

Mountain CF (1986) A new international staging system for lung cancer. *Chest* **89**(suppl.): 225–233

Okazaki T, Takita J, Kohno T, Handa H and Yokota J (1996) Detection of amplified genomic sequences in human small-cell lung carcinoma cells by arbitrarily primed-PCR genomic fingerprinting. *Hum Genet* **98**: 253–258

*© Cancer Research Campaign 1998*

Peinado MA, Sergei M, Velazquez A and Perucho M (1992) Isolation and characterization of allelic losses and gains in colorectal tumors by arbitrarily primed polymerase chain reaction. *Proc Natl Acad Sci USA* **89**: 10065–10069

Perolat P, Merien F, Ellis WA and Baranton G (1994) Characterization of Leptospira isolates from serovar hardjo by ribotyping, arbitrarily primed PCR and mapped restriction site polymorphisms. *J Clin Microbiol* **32**: 1949–1957

Ried T, Petersen I, Holtgreve-Grez H, Speicher MR, Schröck E, Du Manoir S and Cremer T (1994) Mapping of multiple DNA gains and losses in primary small cell lung carcinomas by comparative genomic hybridization. *Cancer Res* **54**: 1801–1806

Rodenhuis S and Slebos RJC (1992) Clinical significance of ras oncogene activation in human lung cancer. *Cancer Res* **52**(suppl.): 2665–2669

Slebos RJC, Kibbelaar RE, Dalesio O, Kooistra A, Stam A, Meijer CJL, Wagenaar SS, Vanderschueren RGJRA, Zandwijk NV, Mooi WJ, Bos JL and Rodenhuis S (1990) K-ras oncogene activation as a prognostic marker in adenocarcinoma of the lung. *N Engl J Med* **323**: 561–565

Sugio K, Kishimoto Y, Virmani AK, Hung JY and Gazdar AF (1994) K-ras mutations are a relative late event in the pathogenesis of lung carcinomas. *Cancer Res* **54**: 5811–5815

Vega FJ, Iniesta P, Caldes T, Sanchez A, Lopez JA, De Juan C, Diaz-Rubio E, Torres A, Balibrea JL and Benito M (1996) Association of K-ras codon 12 transversions with short survival in non-small cell lung cancer. *Int J Oncol* **9**: 1307–1311

Vega FJ, Iniesta P, Caldes T, Sanchez A, Lopez JA, De Juan C, Diaz-Rubio E, Torres A, Balibrea JL and Benito M (1997) p53 exon 5 mutations as prognostic indicator of shortened survival in non-small cell lung cancer. *Br J Cancer* **76**: 44–51

Vogt T, Stolz W, Landthaler M, Ruschoff J and Schlegel J (1996) Nonradioactive arbitrarily primed polymerase chain reaction: A novel technique for detecting genetic defects in skin tumors. *J Invest Dermatol* **106**: 194–197

Weinberg RA (1991) Tumor suppressor genes. *Science* **254**: 1138–1146

Welsh J and McClelland M (1990) Fingerprinting genomes using PCR with arbitrary primers. *Nucleic Acids Res* **18**: 7213–7218

Welsh J and McClelland M (1991) Genomic fingerprinting using arbitrarily primed PCR and a matrix of pairwise combinations of primers. *Nucleic Acids Res* **19**: 5275–5279

WHO (1981) Histological typing of lung tumors. In *International Histological Classification of Tumors*, vol. 1, edn, 2. WHO: Geneva

Yi QM, Deng WG, Xia ZP and Pang-HH (1995) Polymorphism and genetic relatedness among wild and cultivated rice species determined by AP-PCR analysis. *Hereditas* **122**: 135–141

© Cancer Research Campaign 1998


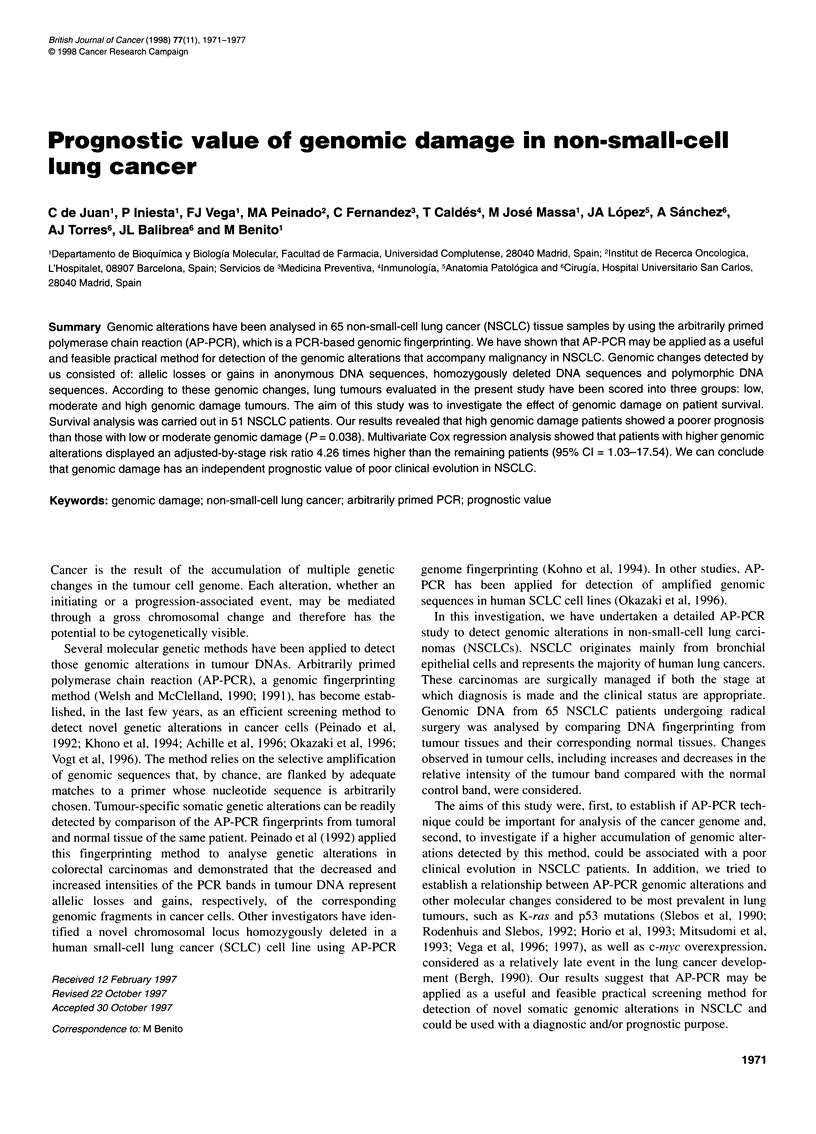

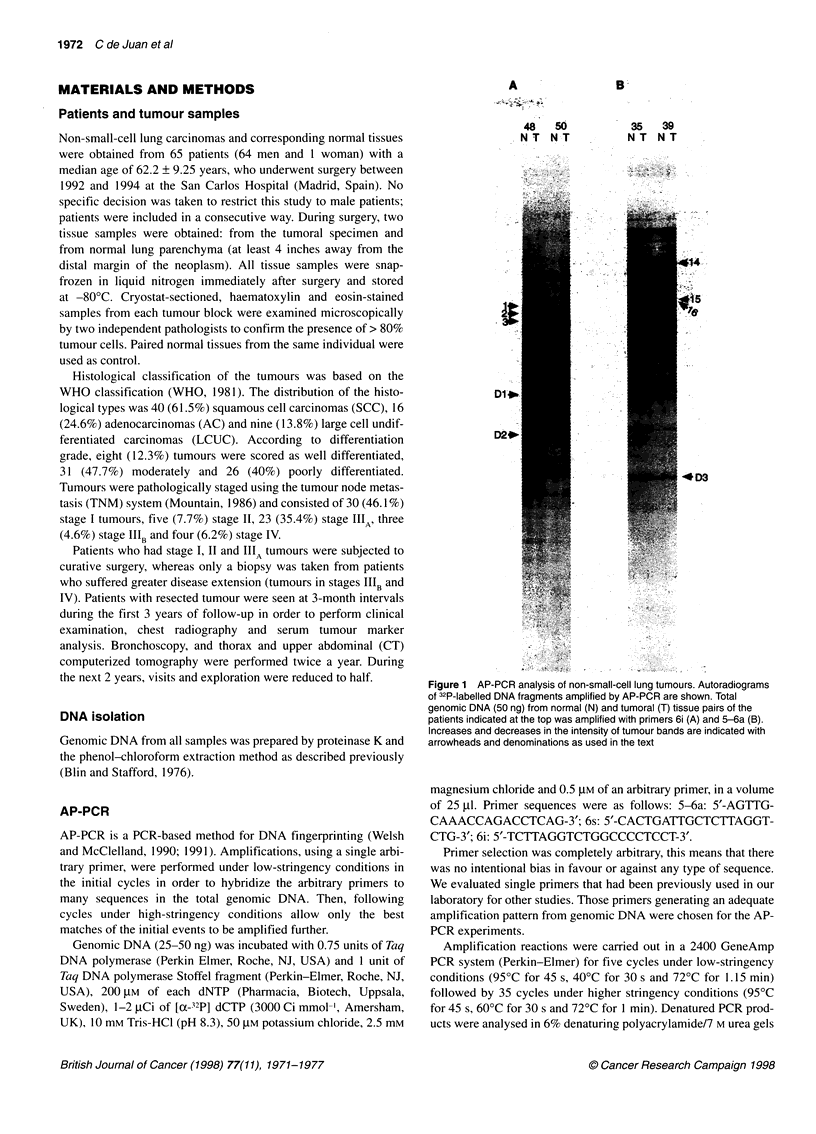

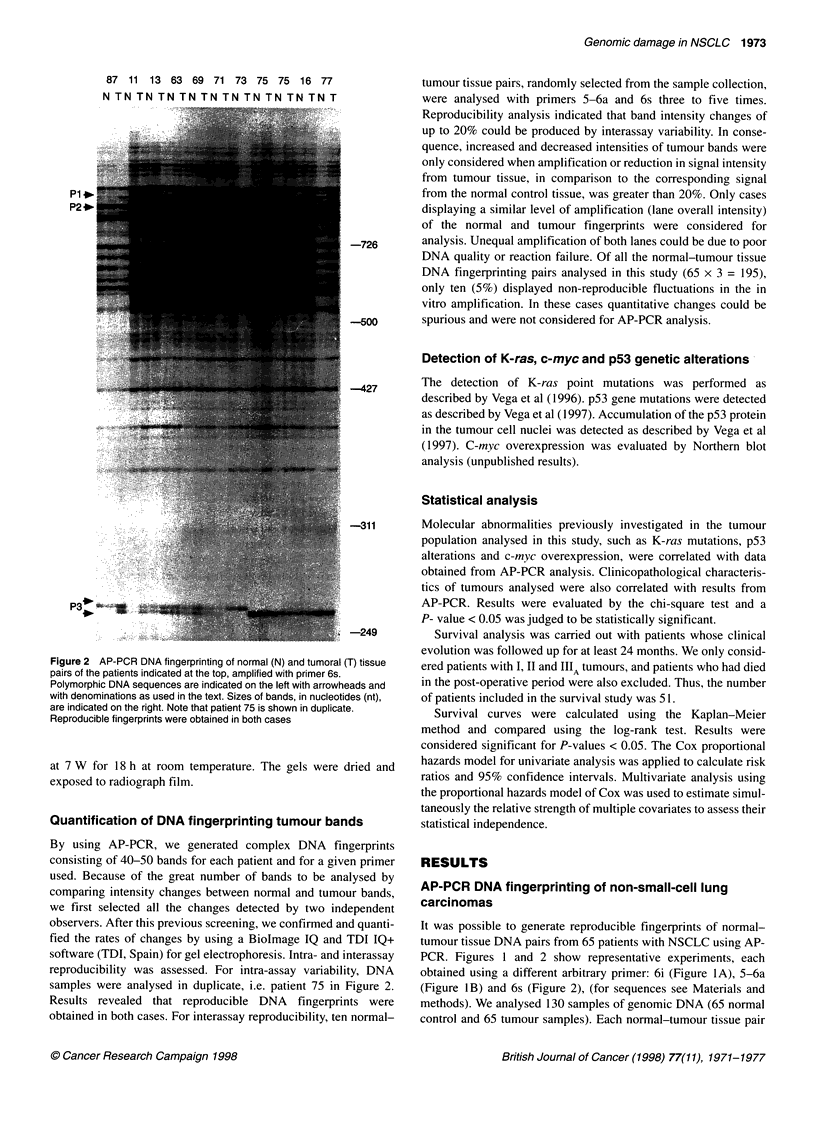

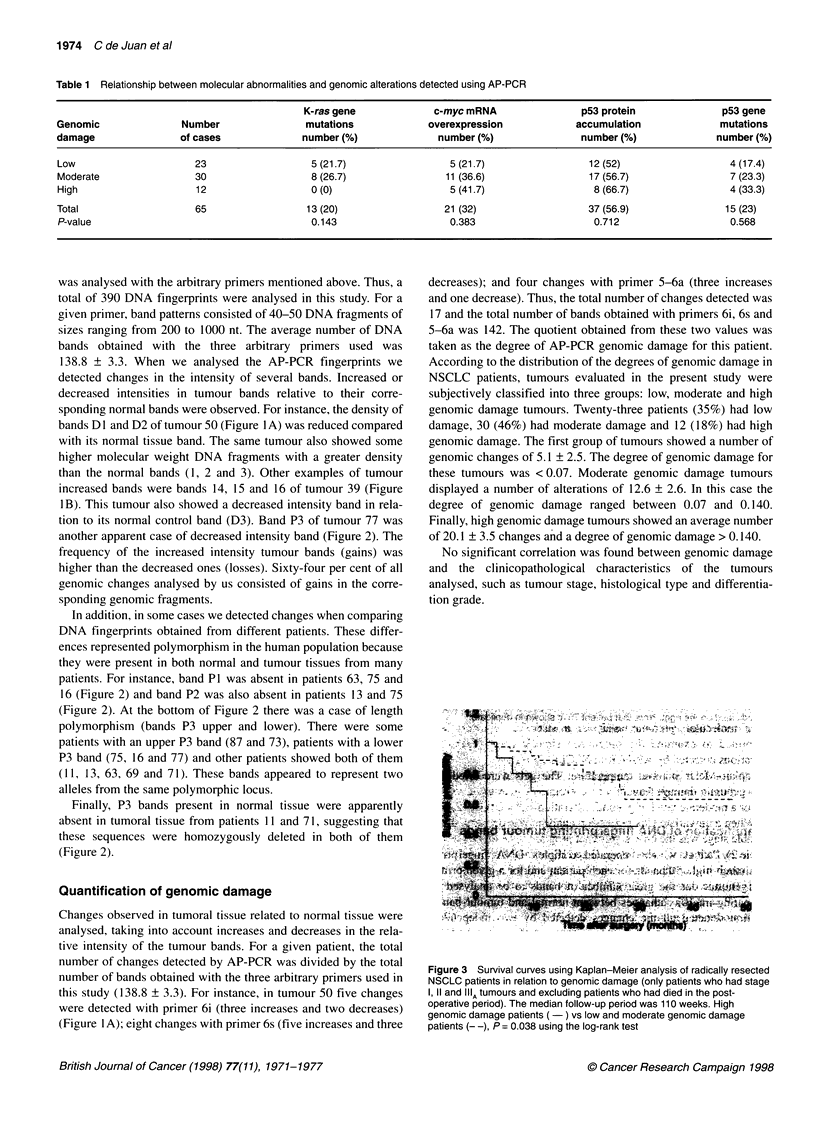

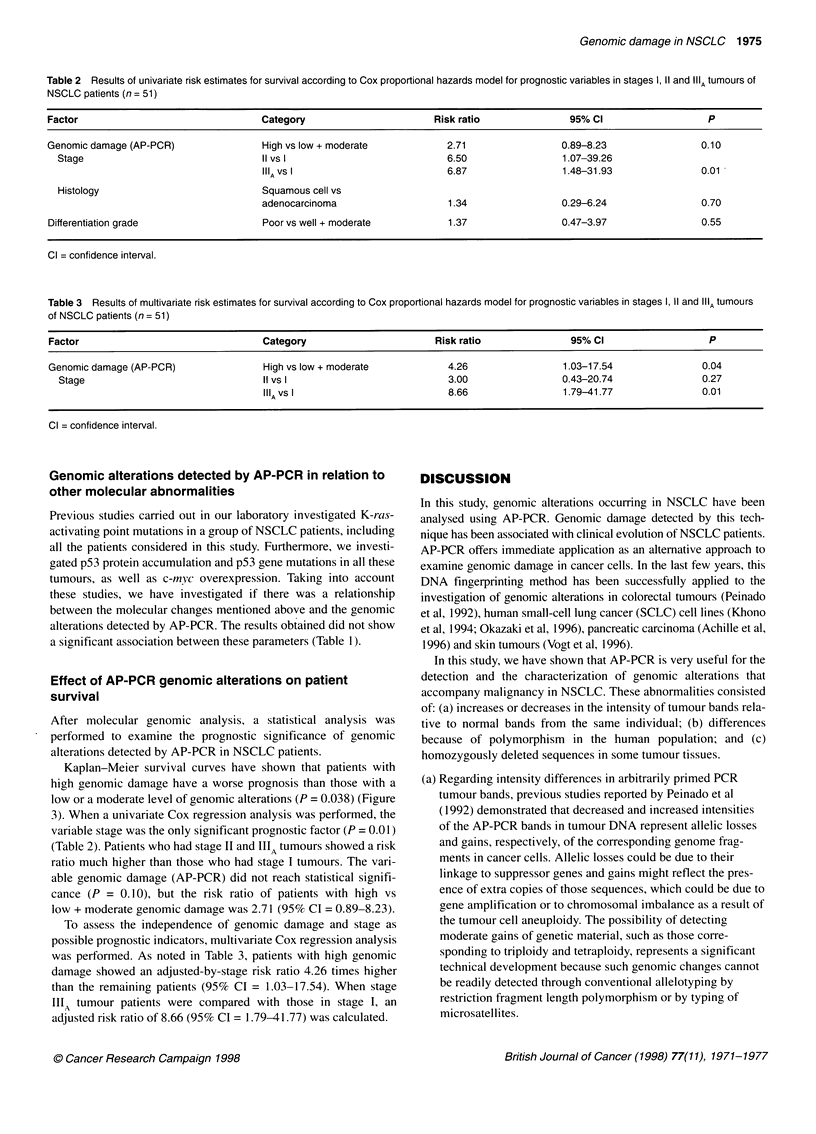

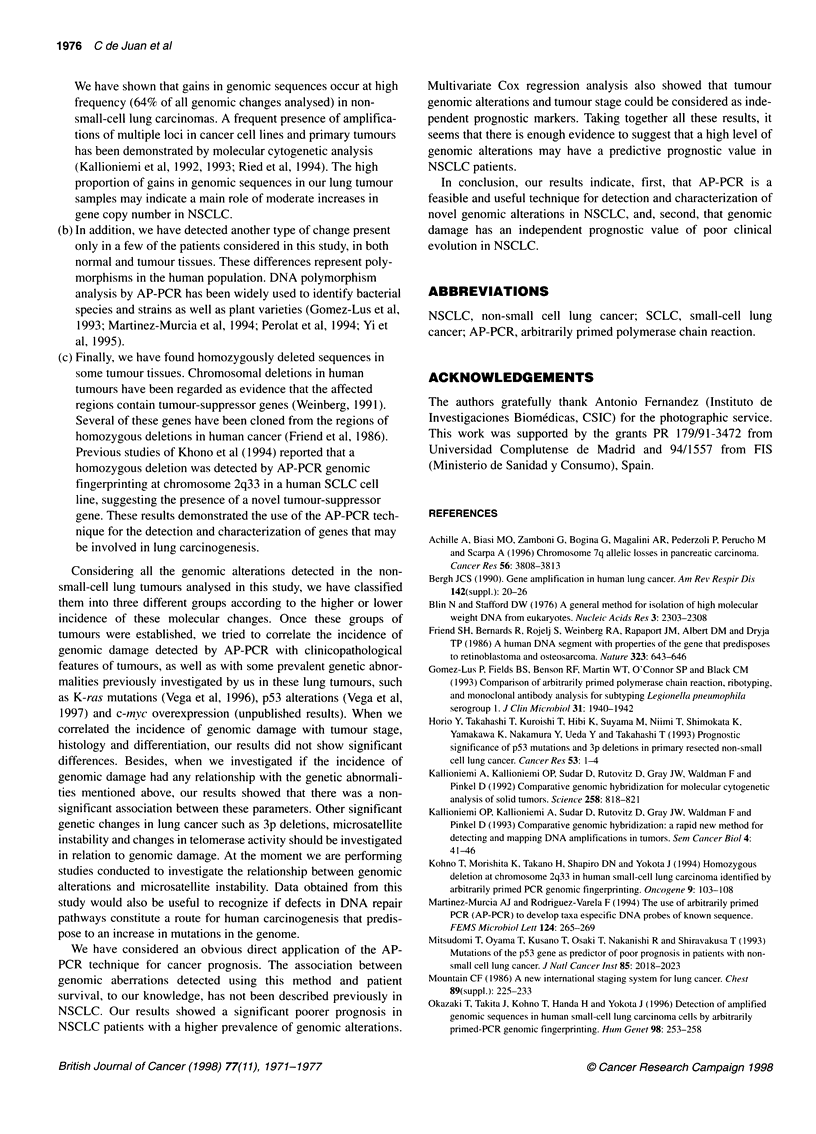

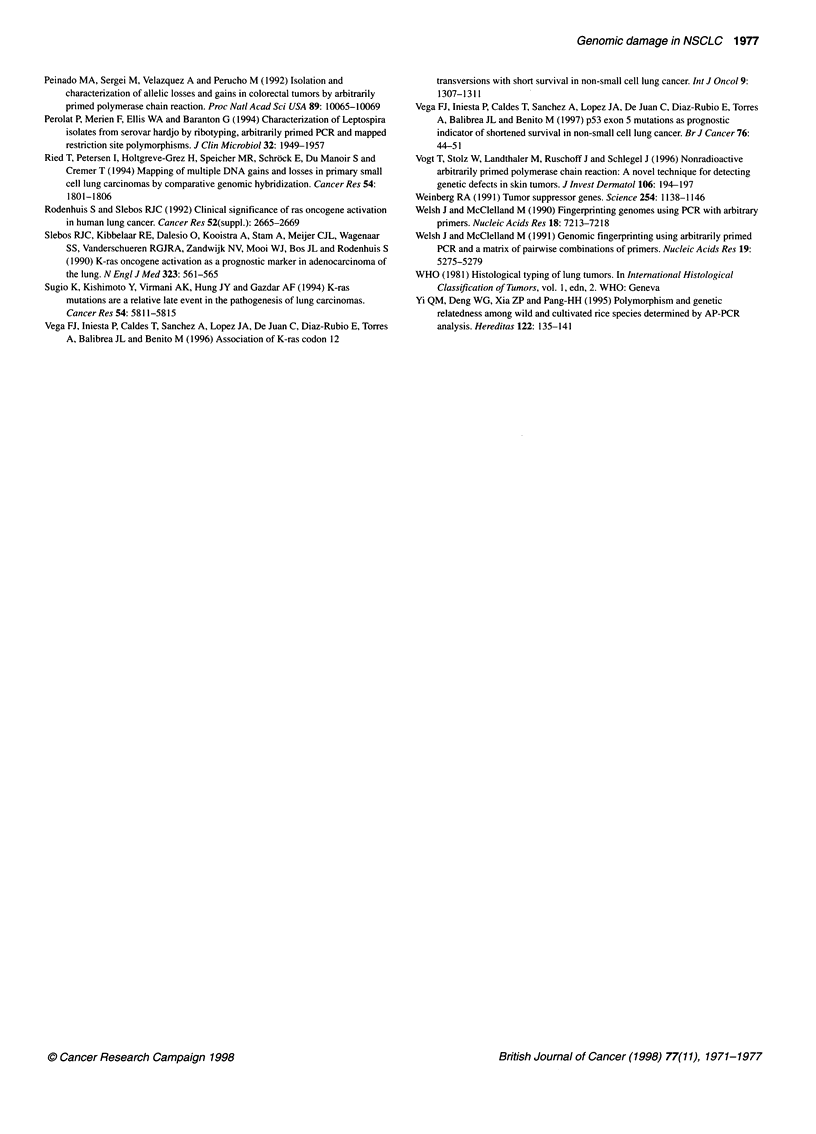


## References

[OCR_00684] Achille A., Biasi M. O., Zamboni G., Bogina G., Magalini A. R., Pederzoli P., Perucho M., Scarpa A. (1996). Chromosome 7q allelic losses in pancreatic carcinoma.. Cancer Res.

[OCR_00693] Blin N., Stafford D. W. (1976). A general method for isolation of high molecular weight DNA from eukaryotes.. Nucleic Acids Res.

[OCR_00697] Friend S. H., Bernards R., Rogelj S., Weinberg R. A., Rapaport J. M., Albert D. M., Dryja T. P. (1986). A human DNA segment with properties of the gene that predisposes to retinoblastoma and osteosarcoma.. Nature.

[OCR_00702] Gomez-Lus P., Fields B. S., Benson R. F., Martin W. T., O'Connor S. P., Black C. M. (1993). Comparison of arbitrarily primed polymerase chain reaction, ribotyping, and monoclonal antibody analysis for subtyping Legionella pneumophila serogroup 1.. J Clin Microbiol.

[OCR_00710] Horio Y., Takahashi T., Kuroishi T., Hibi K., Suyama M., Niimi T., Shimokata K., Yamakawa K., Nakamura Y., Ueda R. (1993). Prognostic significance of p53 mutations and 3p deletions in primary resected non-small cell lung cancer.. Cancer Res.

[OCR_00715] Kallioniemi A., Kallioniemi O. P., Sudar D., Rutovitz D., Gray J. W., Waldman F., Pinkel D. (1992). Comparative genomic hybridization for molecular cytogenetic analysis of solid tumors.. Science.

[OCR_00726] Kohno T., Morishita K., Takano H., Shapiro D. N., Yokota J. (1994). Homozygous deletion at chromosome 2q33 in human small-cell lung carcinoma identified by arbitrarily primed PCR genomic fingerprinting.. Oncogene.

[OCR_00731] Martínez-Murcia A. J., Rodríguez-Valera F. (1994). The use of arbitrarily primed PCR (AP-PCR) to develop taxa specific DNA probes of known sequence.. FEMS Microbiol Lett.

[OCR_00736] Mitsudomi T., Oyama T., Kusano T., Osaki T., Nakanishi R., Shirakusa T. (1993). Mutations of the p53 gene as a predictor of poor prognosis in patients with non-small-cell lung cancer.. J Natl Cancer Inst.

[OCR_00745] Okazaki T., Takita J., Kohno T., Handa H., Yokota J. (1996). Detection of amplified genomic sequences in human small-cell lung carcinoma cells by arbitrarily primed-PCR genomic fingerprinting.. Hum Genet.

[OCR_00754] Peinado M. A., Malkhosyan S., Velazquez A., Perucho M. (1992). Isolation and characterization of allelic losses and gains in colorectal tumors by arbitrarily primed polymerase chain reaction.. Proc Natl Acad Sci U S A.

[OCR_00760] Perolat P., Merien F., Ellis W. A., Baranton G. (1994). Characterization of Leptospira isolates from serovar hardjo by ribotyping, arbitrarily primed PCR, and mapped restriction site polymorphisms.. J Clin Microbiol.

[OCR_00765] Ried T., Petersen I., Holtgreve-Grez H., Speicher M. R., Schröck E., du Manoir S., Cremer T. (1994). Mapping of multiple DNA gains and losses in primary small cell lung carcinomas by comparative genomic hybridization.. Cancer Res.

[OCR_00775] Slebos R. J., Kibbelaar R. E., Dalesio O., Kooistra A., Stam J., Meijer C. J., Wagenaar S. S., Vanderschueren R. G., van Zandwijk N., Mooi W. J. (1990). K-ras oncogene activation as a prognostic marker in adenocarcinoma of the lung.. N Engl J Med.

[OCR_00781] Sugio K., Kishimoto Y., Virmani A. K., Hung J. Y., Gazdar A. F. (1994). K-ras mutations are a relatively late event in the pathogenesis of lung carcinomas.. Cancer Res.

[OCR_00793] Vega F. J., Iniesta P., Caldés T., Sanchez A., López J. A., de Juan C., Diaz-Rubio E., Torres A., Balibrea J. L., Benito M. (1997). p53 exon 5 mutations as a prognostic indicator of shortened survival in non-small-cell lung cancer.. Br J Cancer.

[OCR_00800] Vogt T., Stolz W., Landthaler M., Rüschoff J., Schlegel J. (1996). Nonradioactive arbitrarily primed polymerase chain reaction: a novel technique for detecting genetic defects in skin tumors.. J Invest Dermatol.

[OCR_00805] Weinberg R. A. (1991). Tumor suppressor genes.. Science.

[OCR_00807] Welsh J., McClelland M. (1990). Fingerprinting genomes using PCR with arbitrary primers.. Nucleic Acids Res.

[OCR_00811] Welsh J., McClelland M. (1991). Genomic fingerprinting using arbitrarily primed PCR and a matrix of pairwise combinations of primers.. Nucleic Acids Res.

[OCR_00820] Yi Q. M., Deng W. G., Xia Z. P., Pang H. H. (1995). Polymorphism and genetic relatedness among wild and cultivated rice species determined by AP-PCR analysis.. Hereditas.

